# An update on thermostable keratinases for protein engineering against feather pollutants

**DOI:** 10.1007/s00253-025-13459-5

**Published:** 2025-03-25

**Authors:** Bhagya Jyothi J L, Immanuel Dhanasingh

**Affiliations:** Centre for Bio-Separation Technology, Vellore Institute of Technology, Vellore, Tamil Nadu 632014 India

**Keywords:** Feather-pollutant, Keratinase, Thermophile, Enzymatic keratinolysis, Protein-engineering, Bioremediation

## Abstract

**Abstract:**

Every year, the poultry business worldwide produces at least 8.5 billion tonnes of chicken feathers, making it one of the major landfill pollutants in the world. Biodegradation and recycling of native feathers is difficult due to the presence of numerous disulfide linkages in the feather’s major constituent, keratin. Denaturation of such recalcitrant protein is thermodynamically favored at high temperatures. Therefore, the lookout for the enzymes that degrade keratin (keratinases) from thermophilic bacteria resulted in the identification of thermostable enzymes favoring feather degradation at high temperatures. This review presents a comprehensive analysis of the biochemical properties and structural attributes of thermostable keratinases, emphasizing their catalytic mechanisms, stability at high temperatures, and substrate specificity. Our exploration of structural features enables us to understand the molecular architecture of these enzymes for protein engineering that might enhance the keratinolytic activity and thermostability further. As the field of protein engineering advances, there exists a pressing requirement for integration of structural data with pragmatic engineering applications. Our review addresses for the first time the detailed structural aspects of thermostable bacterial keratinolytic enzymes that will facilitate the development of modified keratinases through protein engineering for a broad range of industrial applications, such as in the production of biofuels, leather processing, and waste management.

**Keypoints:**

*• Efficient eco-friendly bioremediation of feather landfill pollutant using thermophilic keratinases.*

*• Detailed structural and biochemical aspects of different thermophilic bacterial keratinases.*

*• Combinations of thermostable keratinases for the enhanced feather degradation process*

**Graphical Abstract:**

Feather waste degradation using bacterial keratinases: an eco-friendly bioprocess for degradation of keratin-rich feather wastes into nutrient-rich byproducts, biofertilizers, and animal feed, using bacterial keratinases. A recycling strategy, contributing to pollutant degradation and waste management.

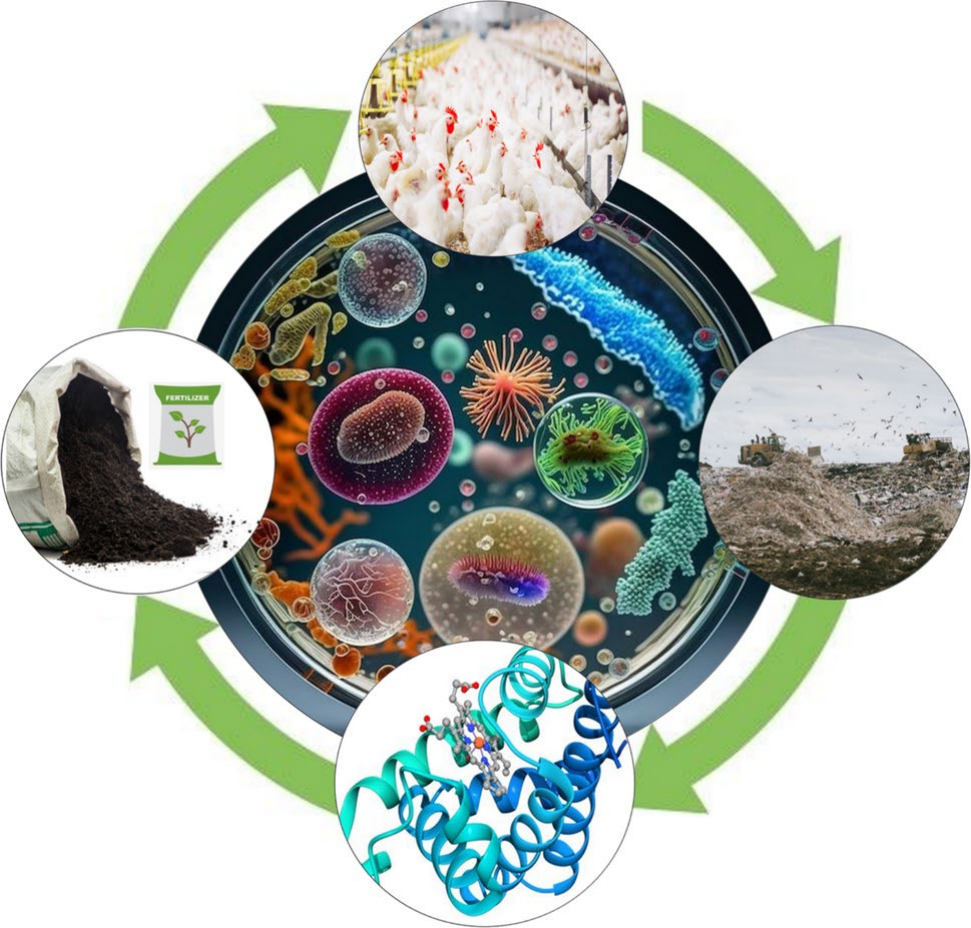

## Introduction

The global poultry industry generates an immense amount of waste annually, with feathers constituting a significant waste byproduct, comprising approximately 5–7% (w/w) of the total weight of an adult chicken (Manczinger et al. 2003). Due to the recent rise in the world’s consumption of poultry meat, an enormous quantity of natural feathers is continuously being released as an environmental solid waste during the processing of poultry (Kang et al. 2020). It is estimated that approximately 8.5 billion tonnes of poultry feathers are disposed of on a global scale annually, with India solely accounting for an estimated 350 million tonnes each year (Nad et al. 2024; Kumari et al. 2020). However, there is a significant challenge associated with the management of these feather-containing poultry wastes due to their non-biodegradable characteristic that poses a notable environmental threat (Brandelli et al. 2015). This recalcitrant nature of feather is mainly due to a major constituent fibrous protein named keratin. Keratin not only renders the feathers insoluble, but also makes them resistant to common proteolytic enzymes such as pepsin and trypsin. The sturdiness of keratin is due to its stable structure, stabilized by numerous disulfide bridges, intra- and inter-molecular hydrogen bonds, and hydrophobic interactions (Jain et al. 2012; Mehra et al. 2022). The natural ability of feathers to resist decomposition leads to their accumulation as a landfill for a long period of time. This accumulation not only results in the pollution of soil and water but also promotes the dissemination of various human ailments, including dermatophytic infections, chlorosis, mycoplasma fowl cholera, and avian influenza (Jain et al. 2012). Therefore, the waste management of the feather continues to persist as a global menace in the context of successful bioremediation.

To develop an efficient feather degradation system, it is prerequisite to understand the main constituent, keratin, that provides tenaciousness to feathers. The keratin’s mechanical stability and resistance to degradation are determined by the tight packing of polypeptide chains either in α-helix (α-keratin) or β-sheet (β-keratin) structures (Yamamura et al. 2002; Rodman et al. 2018). On average, whole feathers tend to have 32.2% α-helix, 53.6% β-sheets, and 14.2% turns in random coils (Qiu et al. 2020). Alpha (α) keratin is characterized by a high prevalence of hydrophobic amino acids, including methionine, phenylalanine, valine, isoleucine, and alanine, while beta (β) keratin is abundant in glycine, lysine, histidine, alanine, serine, and tryptophan. (Varanko et al. 2020). In addition, β-keratin has a substantial content of cysteine (4–20%), that rapidly forms disulfide bonds, conferring high rigidity and improved resistance to degradation (Alibardi 2016; Suh et al. 2001). Thus, the feather with β-keratin as major constituent is resilient to degradation due to high cysteine content and disulfide bridges conferred by them (Greenwold et al. 2014; Feroz et al. 2020). On the other hand, the same keratin constituting nearly 90% of feather composition may serve as a viable source of proteins and amino acids for livestock, if bioremediated efficiently (Jain et al. 2012; Sim et al. 2023). Furthermore, keratin is unique in that it contains relatively high levels of sulfur due to sulfur-containing amino acids including cystine, cysteine, and methionine, and nitrogen from the amino-group of amino acids (Sharma et al. 2011). Due to the presence of these micronutrient components in abundance, feather meal can be a substantial reservoir of nutrients for both plants and animals, serving as fertilizers and animal feeds, respectively (Srivastava et al. 2020; Wainwright et al. 1985; Korniłłowicz-Kowalska et al. 2011).

**Fig. 1:**
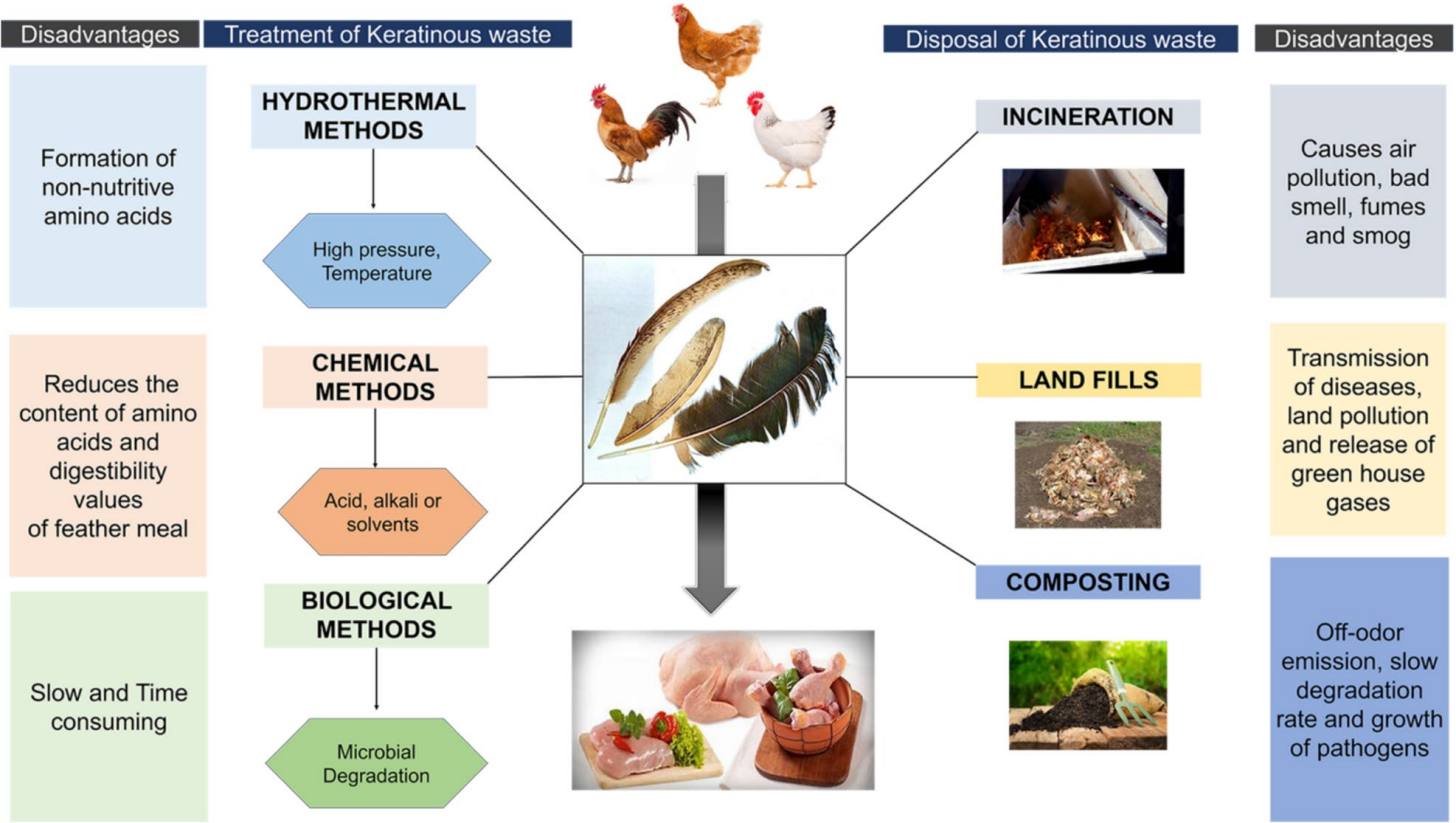
Conventional methods of feather waste management and their drawbacks

Traditionally, the proteolytic resistant feather wastes were either incinerated, deposited in landfills, or chemically broken down (Fig. 1), resulting in various adverse consequences such as: (i) destruction of essential amino acids, resulting in a reduction in the quality and digestibility of protein in nutrient-rich feathers (Dipankar et al. 2019; Pandey et al. 2019), (ii) contribute to environmental pollution and a variety of human health issues (Pandey et al. 2019), (iii) production of inferior feather meal with respect to nutrient loss upon acid hydrolysis (Jain et al. 2012; Cheong et al. 2018), and (iv) risk of nitrate seepage into groundwater, phosphorus runoff into adjacent water sources, and a potential surge in bacterial or viral diseases due to large landfills (Bhari et al. 2021). These critical issues lessened the usage of traditional feather disposal systems (Fig. 1) and instead compelled the search for more efficient and eco-friendly feather waste treatment methods.

Decades of research have revealed a viable solution for feather degradation via microbial keratinolysis, that can prevent the destruction of nutrients and lessen environmental pollution. Microbial keratinolysis is a process in which keratin from feather can be degraded using keratinase enzymes produced by certain microorganisms (Manczinger et al. 2003; Kim 2007). The discovery of such feather-degrading microorganisms was critical and revolutionary in the field of feather bioremediation (Li 2019) (Table 1). Numerous feather-degrading bacteria (FDB) have been isolated from various genera, including *Bacillus, Staphylococcus*, *Enterococcus*, *Streptomyces*, *and Pseudomonas* sp., among others (Li et al. 2020) (Table 1). The majority of mesophilic FDB require nearly ≥ 5–6 days to fully decompose just 1% feather-containing medium (Li et al. 2020). Despite microbial keratinolysis being an eco-friendly option, the problem is that the feather-degrading capacity of mesophilic microbes decreases at extreme temperature and pH conditions during large-scale treatment at industrial conditions. This proved to be very inefficient in the degradation of several tonnes of feathers produced every day in the poultry industries (K. Zhang and Han 2024; Peng et al. 2022). Therefore, numerous extremophilic feather-degrading microbes were screened that can have a greater keratinolytic efficiency (Ghasemi et al. 2012) (Table 1). Accordingly, several thermophilic and hyperthermophilic feather-degrading bacteria such as *Thermoanaerobacter keratinoplilus*, *Thermoanaerobacter* sp. strain 1004–09, *Fervidobacterium pennivorans*, *F. islandicum* AW-1, *F. thailandense* FC2004T, and *Fervidobacterium* sp. FA004 were identified (Lee et al. 2015b). Friedricht and Antranikian 1996; Nam et al. 2002; Kanoksilapatham et al. 2016). These had a remarkable feather-degrading capability of degrading the same feathers within 48–72 h at temperatures greater than 60 °C, making them industrially more significant (Intagun and Kanoksilapatham 2017) (Table 1).

**Table 1:** Identified keratin-degrading bacteria and their optimal conditions for activity

Sl no	Bacteria sp.	Keratinolytic activity (in units)	Optimum temperature (°C)	Optimum pH	References
1	*Bacillus licheniformis* K19	224 U/ml	60	7.5–8	(Xu et al. 2009)
2	*Bacillus* sp. MKR5	225U/ml	70	8	(Ghasemi et al. 2012)
3	*Bacillus* sp. CSK2	1539.09 U/ml	60–80	8	(Nnolim and Nwodo 2020)
4	*Chryseobacterium* sp. RBT	98 U/ml	50	8.6	(Gurav and Jadhav 2013)
5	*Paracoccus* sp. WJ-98	90 U/ml	50	6.8	(Lee et al. 2004)
6	*Nocardiopsis* sp. SD5	64.66 U/ml	50	9	(Saha et al. 2013)
7	*Streptomyces gulbargensis*	1.39 U/ml	45	9	(Syed et al. 2009)
8	*Bacillus* sp. MKR5	225 U/ml	40	8	(Ghasemi et al. 2012)
9	*Streptomyces* sp. S.K1–02	341 U/mg	70	10	(Letourneau et al. 1998)
10	*Caldicoprobacter algeriensis*	21,000 U/ml	50	7	(Bouacem et al. 2016)
11	*Bacillus cytotoxicus* LT-1	17.1 U/ml	50	7	(Cavello et al. 2020)
12	*Bacillus halodurans* SW-X	16.05 ± 0.25 U/mL	47	10	(Kaewsalud et al. 2021)
13	*Fervidobacterium islandicum* AW-1	NA	70	7	(Nam et al. 2002)
14	*Thermoactinomycetes* sp. YT06	1325 U/mg	65	8.5	(Wang et al. 2019)
15	*Bacillus subtilis* NRC 3	169 U/mg	40–50	7.5–8	(Tork et al. 2013)
16	*Chryseobacterium* sp. kr6	967U/mg	50	8.5	(Riffel et al. 2007)
17	*Pseudomonas aeruginosa* K13	280U/mL	40	7–8	(Patil and Jadhav 2017)
18	*Bacillus* sp. NDS-10	NA	65	7–8	(Akram et al. 2021)
19	*Bacillus* sp.	681U/mL	40	8	(Selvankumar et al. 2018)
20	*Bacillus* sp. MKR5	225 U/mL	70	8	(Ghasemi et al. 2012)
21	*Deinococcus geothermalis*	NA	70	9–11	(Tang et al. 2021)
22	*Deinococcus gobiensis*	51,147 U/mg	60	7.5–9.0	(Meng et al. 2022)
23	*Streptomyces enissocaesilis* AM1	0.032 U/mL	50	7	(Khalel 2020)
24	*Anoxybacillus caldiproteolyticus* PC2	28.3 ± 11.5 U/mL	50–60	7	(Reis et al. 2020)
25	*B. velezensis* NCIM 5802	109.7 U/mL	60	10	(Sharma et al. 2022)

*NA*, data not available

Despite these advances with microbial degradation, there still exists a time lag to destroy several tonnes of feathers produced every day due to lagging efficiency in terms of microbial keratinolysis. In addition, the resources spent on bacterial growth, media preparation, bioreactor condition, and sterility maintenance in order for them to destroy feathers, are expensive and strenuous. Therefore, with the advances made in the field of enzymology, the keratin-degrading enzymes (keratinases) from the bacteria can be overexpressed and isolated using recombinant technology, and used industrially for recycling feather waste into a nutrient feed. These advances put forth enzymatic keratinolysis ahead of microbial feather degradation.

**Fig. 2:**
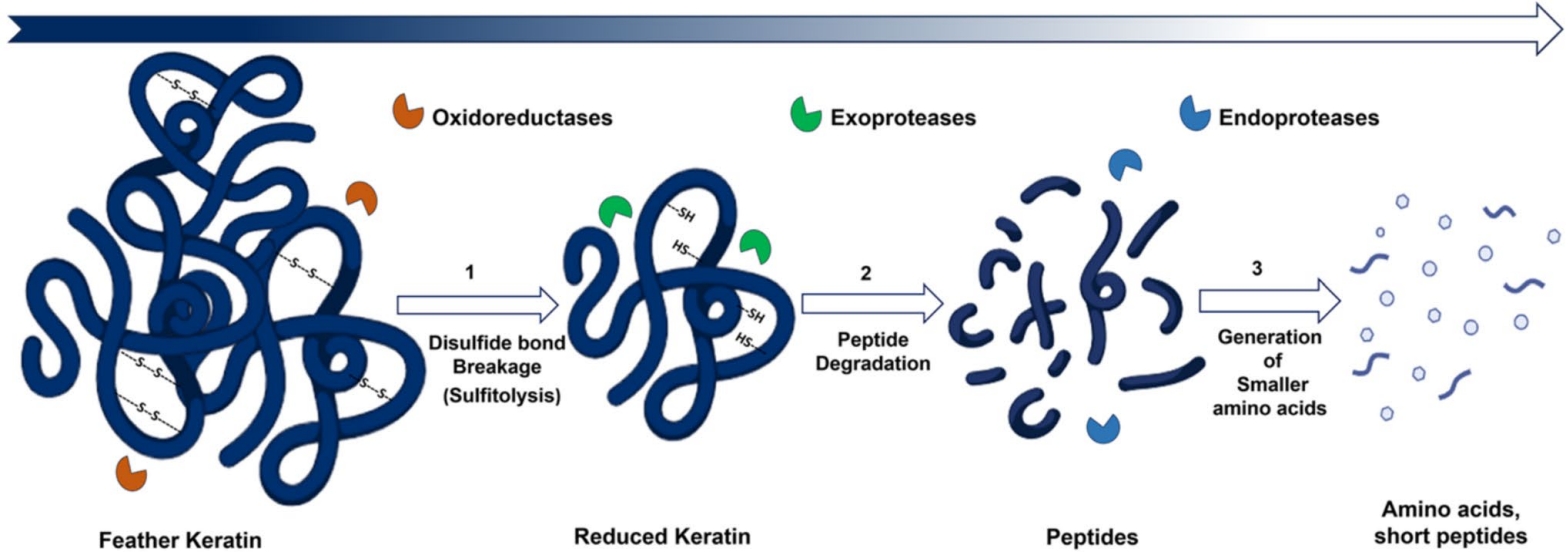
Mechanism of microbial keratinase-mediated feather degradation: (1) reduction of numerous disulfide bridges by reductase enzymes; (2) hydrolysis of peptide bonds by exo-proteases that generate multiple peptide fragments; and followed by (3) release of free amino acids by action of endo-proteases (created with Biorender.com)

Accordingly, several research groups were focused on identifying the keratinases present in these feather-degrading microbes that are critical for feather degradation and utilization ex vivo (Kang et al. 2020; Z. W. Li et al. 2020). The identified keratinases were a family of exo- and endo-proteases from keratin-degrading microorganisms with a unique ability to depolymerize fibrous, resistant structural keratins into soluble proteins through keratinolytic activity (Qiu et al. 2020; I. Sharma et al. 2022; M. Sharma et al. 2011). Studies have further revealed that there are multiple enzymes present in the feather-degrading bacteria that coherently are involved in different steps of keratinolysis (Table 2; Fig. 2). First, certain unidentified reductases from the FDB induce reduction of disulfide bridges (-S–S-) present in feather keratin; second, exo- and endo-proteases are involved in the hydrolysis of peptide bonds from the reduced feather that facilitate the breakdown of feather keratin (Kang et al. 2020). The identification and isolation of these keratinases pave way to the establishment of an ex vivo enzymatic feather degradation system.

**Table 2:** Thermostable keratinases identified in different bacterial species that are characterized

Sl. no	Species	Keratinases identified	References
1	*Bacillus* sp*.* CN2	γ- Glutamyl transferase (GGT), S8B serine endopeptidase, M4 metalloprotease, S8C, and S8A serine proteases	(Lai et al. 2023)
2	*Bacillus subtilis* CH1	Extracellular protease Vpr, glyoxal/methylglyoxal reductase (YvgN), γ-glutamyl transpeptidase	(Chen et al. 2020)
3	*Bacillus* sp. 8A6	M12 family (metalloproteases), serine proteases (S01A, S08A family), T3 family (γ-glutamyltransferases), and oligopeptide- and dipeptide-binding proteins	(Huang et al. 2020)
4	*Fervidobacterium islandicum* AW-1	β-Aspartyl peptidase (*Fi*BAP), M32 carboxypeptidase (*Fis*CP), ATP-dependent serine protease, sulfur formation system (suf system), pyrrolidone carboxypeptidase, metalloproteases	(La et al. 2020; Yong Jik Lee et al. 2015a, b; Dhanasingh et al. 2021; Jin et al. 2021; Kang et al. 2020)
6	*Streptomyces* sp. SCUT-3	Proteinases: SCUT-3-Sep39, SCUT-3-Sep40, SCUT-3-Sep53	(Lu et al. 2024; Z. W. Li et al. 2020)
7	*Streptomyces* sp. G11C	Serine proteases (S01, S08 family), metallo proteases (M04, M06 family)	(Valencia et al. 2021; González et al. 2020)

Keratinase enzymes that are derived from thermophiles and hyperthermophiles called thermozymes, with notable properties such as being active at high temperatures, having heat stability, and being resistant to denaturing conditions such as solvents and detergents (Kang et al. 2020) (Table 2). The stability and activity of thermozymes have proven to be remarkable within the temperature range of 60 to 125 °C, transcending the tolerance of mesophilic enzymes (Vieille and Zeikus 1996). With thermostability being a critical factor in industrial settings, keratinolytic thermozymes might be a critical and viable solution towards feather degradation (Liyuan Wang 2019; Tadevosyan et al. 2022). Furthermore, the application of novel genetic engineering methods, such as genetic code expansion and site-directed mutagenesis, have led to the creation of thermostable keratinase variants that demonstrate increased keratinolytic efficiency (Gahatraj et al. 2023; Q. Li 2021; Wu et al. 2017). Accordingly, to re-engineer proteins, it is imperative to understand the structural attributes of already known thermostable keratinases, including their active site and residues contributing to thermostability and keratinolytic activity, in order to effectively generate the next-generation keratinolytic thermozymes that can withstand challenging industrial environments characterized by elevated temperatures and fluctuating pH levels.

This review would provide a comprehensive update on the critical structural features of five thermostable keratinases which can be manipulated and utilized in industrial processes for efficient conversion of feathers into feed and fertilizers. Also, the comprehension of the structure and biochemistry of keratinases enhances their utilization in several applications, such as the pharmaceutical, leather, and textile sectors. Although microbial feather degradation is a field that has been explored over several decades with numerous review articles, here, we have focused in detail on the structural and protein engineering aspects of thermostable keratinases that might show improved feather degradation efficiency. Our analysis on the current thermostable keratinases might pave the way for the development of next-generation thermostable re-engineered keratinases, applicable to all keratin-related waste management industries.

## Keratin-degrading microbes: an overview

A number of microorganisms, such as bacteria, actinomycetes, and fungi, possess the ability to utilize keratins from feathers as carbon, nitrogen, sulfur, and energy sources to sustain their growth, referred generally to as keratinolytic microbes (de Menezes et al. 2021; Hassan et al. 2020).

The majority of keratinolytic microbes are derived from bacterial sources (Table 1), with *Bacillus* genera being the most prevalent, which include *Bacillus subtilis*, *Bacillus pumilus*, *Bacillus lichenifomis*, *Bacillus paralichenifomis,* and *Bacillus cereus* (Tamreihao et al. 2019). Whereas, *B. licheniformis* PWD-1, *Streptomyces thermonitrificans* MG104, *Meiothermus ruber* H328, *Meiothermus* sp. I40, *Bacillus subtilis* (*B. subtilis*) RM-01, *Brevibacillus thermoruber* T1E, *B. halodurans* JB99, and *Thermoactinomyces* sp. YT06 are instances of keratinolytic bacteria that can be classified as moderately thermophilic aerobic bacteria, particularly the ones that thrive in temperatures ranging from 50 to 60 °C (Cavello et al. 2020). Furthermore, a number of hyperthermophilic and thermotolerant keratinolytic bacteria that survive at > 80 °C have been isolated from hot volcanic regions, which include *Fervidobacterium pennivorans*, *F. islandicum* AW-1, *Thermoanaerobacter keratinophilus* 2KXI, *Caldanaerobacter* sp. strain 1523–1, and *Clostridium sporogenes* bv. pennivorans.

The significant thermophilic strains of keratinolytic actinomycete species, namely *Streptomyces albidoflavus*, *S. fradiae*,* S. graminofaciens*,* S. pactum*, and *Hermoactinomyces candidus*, were utilized in the production of keratinase using various keratin substrates (Hassan et al. 2020). Particularly, *S. rochei* AM8 and *S. netropsis* have been studied for their feather-degrading capabilities, with recorded keratinase activities of 0.782 U/mL (one unit (U)/mL refers to the enzyme quantity (μM) that liberates 1 micromole (μmol) of product per mL of the reaction mixture per minute) and 113.6 U/mL, respectively (Alamoudi et al. 2022; Abdelmoteleb et al. 2023). Actinomycetes, such as *Thermoactinomyces* sp. YT06, synthesize keratinases that exhibit enzymatic activity across a broad spectrum of pH levels and thermal conditions, thereby augmenting their applicability in numerous industrial contexts (Wang et al. 2017). For instance, the keratinolytic activity of certain actinomycetes, like *Streptomyces* sp. TBG-S13A5, has been effectively utilized in various applications, including the dehairing and destaining of leather, thereby demonstrating the multifaceted nature of these enzymes within industrial methodologies (Indhuja et al. 2012). Marine ecosystem-derived actinobacteria also display keratinolytic capabilities, with specific strains showing remarkable stability and performance under various pH and temperature conditions (Avdiyuk et al. 2021).

Fungi that degrade keratin can be obtained from many tissues of both humans and animals, as the keratinase they produce potentially plays a significant role in fungal infections (Li 2021). The predominant keratinolytic group of fungi comprises *Chrysosporium indicum*, *C. evolceanui*, *Trichophyton terrestre*, *C. lobatum*, *Microsporum gypseum*, *Acremonium strictum*, *Acremonium* sp., *Malbranchea* sp., *Microsporum gypseum*, *Aspergillus candidus*, *T. simii*, *Chrysosporium keratinophilum*, *C. pannicola*, *C. anum*, *M. canis*, *and M. fulvum*, which are primarily obtained from soils found in feather dumping sites or chicken farms (Srivastava et al. 2020). Several *Aspergillus* species, including *Aspergillus terreus*, *Aspergillus nidulans, Aspergillus niger*, and *Aspergillus fumigatus* with keratinolytic activity, were cultivated using solid-state fermentation and demonstrated significant potential in feather waste management (Derhab et al. 2022). *Aspergillus niger*, for instance, has been recognized for its high protease enzyme activity, which is crucial for keratin degradation (B. Singh et al. 2022). Similarly, *Chrysosporium tropicum* and *Aphanoascus keratinophilus*, isolated from bird of prey pellets, showed substantial keratinolytic activity, with feather weight loss ranging from 55 to 75% (Bohacz et al. 2020). *Talaromyces sayulitensis* GF11, a recently characterized strain, has demonstrated encouraging efficacy in the degradation of keratin derived from chicken feathers (Sutoyo et al. 2019).

Despite the availability of a wide diversity of keratinolytic microbes, major industrial and research focus remains preferably on bacterial keratinases due to their efficiency, stability, versatility, and industrial applicability. Consistently, studies have demonstrated that bacterial species, specifically *Bacillus subtilis*, are capable of attaining optimal activity in keratinolysis within a reduced time frame relative to fungal organisms such as *Aspergillus fumigatus*, which necessitates an extended duration to achieve comparable degrees of decomposition (Sivakumar and Raveendran 2015). Furthermore, isolation and purification of fungal keratinase enzymes present greater difficulties and expenses than bacterial keratinases, which is attributed to the extracellular characteristics of numerous fungal enzymes, complicating downstream processing (Chaudhary et al. 2021; Moonnee et al. 2021; Bhari and Kaur 2023). Furthermore, the adaptable culture and growth conditions of bacteria that facilitate efficient enzyme production and application make them a suitable choice for industrial preference (Karaveli and Deniz 2021). In contrast, microbes like fungi typically require more extensive growth conditions, such as complex optimization of culture media and bioreactor designs for mass and heat transfer, limiting their application. These indications clearly support the superior advantages of using bacterial keratinases rather than fungal or actinomycetes keratinases on an industrial scale.

## Mechanism of feather degradation

As mentioned earlier, the feather degradation is severely hampered by the recalcitrant nature of its major component, the keratin. The entire sequence of reactions associated with keratin degradation remains substantially ambiguous, with only a few hypotheses proposed (Jin et al. 2021; Lai et al. 2023). In general, bacterial keratinases work through a series of synergistic enzymatic reactions to break down the complex structure of keratin into smaller, more manageable fragments (Li 2021) (Fig. 2; Table 2).

Any microbial keratinolysis process in general involves first the reduction of numerous disulfide bridges in keratin. This is performed by several unidentified reductase enzymes from the feather-degrading bacteria (FDB) (Fig. 2). Disulfide reductases or reducing agents are two possible catalysts for reduction reactions (Li 2021). Disulfide reductases catalyze sulfitolysis by the cleavage of β-sheet disulfide bonds, and the process requires the presence of living cells or reductants like glutathione, mercaptoethanol, cysteine, dithiothreitol, sodium sulfite, or thioglycolate to break down keratin into a partially chopped protein by attacking the strong disulfide bonds between two residues (Srivastava et al. 2020). Free cysteinyl groups also play a significant role as reducing agents in feather sulfitolysis, in addition to sulfite (Li et al. 2020). The process of sulfitolysis releases thiol groups by cleaving disulfide connections between polypeptide keratin chains. In the presence of sulfite, disulfide bonds are cleaved to generate cysteine and S-sulfocysteine (Srivastava et al. 2020). Reduction of disulfide bonds changes the conformation of amino acids in the β-sheet of keratin, rendering different hydrolytic sites for proteolytic attack by keratinases that result in the release of amino acids and soluble peptides (Tamreihao et al. 2019). It has also been proposed that the cysteine-cystine cycle between FDB and the extracellular feather environment might also be involved in the sulfitolysis of the keratin protein (Jin et al. 2021).

Second, upon disulfide reduction, the feather is accessible to several exo-proteases, such as S8 exopeptidases, that hydrolyze the peptide bond, thereby generating multiple peptide fragments (Lai et al. 2023; Kang et al. 2020) (Fig. 2; Table 2). The serine protease S8 exopeptidases, recognized for their extensive substrate specificity and capacity to operate in conjunction with other proteolytic enzymes, facilitate the attack on keratin, thereby hydrolyzing at the terminal ends of the protein (Qiu et al. 2020; Zhou et al. 2023). Similarly, beta-aspartyl peptidases selectively target the peptide bonds that are proximal to iso-aspartic acid residues (W. Aehle et al. 2009), which are abundant within keratin structures and play a significant role in the hydrolysis of keratin (Kang et al. 2020). The generated peptides are then subjected to the next level of keratin-degrading enzymes.

Third, the generated peptide fragments are either transported into the bacteria or disintegrated further outside by means of endo-peptidases such as M32 carboxypeptidase or exo-proteases, respectively, releasing multiple amino acids, which are the final products of keratinolysis (Qiu et al. 2020; M. Kuddus 2017) (Fig. 2; Table 2). In addition to the hydrolyzed amino acids, proteolytic activity linked to sequential deamination activities of the amino acids releases surplus of nitrogen in the form of ammonium ions, which are valuable constituents in animal feed and fertilizers (Tamreihao et al. 2019). Through these means, the complex feather keratin hydrolyzes to simpler amino acids and peptides (Fig. 2) that are suitable for multiple applications.

## Thermostability: a crucial property in feather degradation

The temperature at which a certain enzyme fastens the reaction rate is dependent on the optimal growth condition of that particular organism from which the enzyme is derived. For example, the enzymes from mesophilic bacteria have an optimal temperature in the range of 25–40 °C, while the enzymes from thermophiles can act at an optimal temperature as high as 60–80 °C. For any enzymatic reaction, with rising temperatures, the activation energy required for enzymatic activity decreases. Use of thermostable enzymes such as L-arabinose isomerase (L-AI) from *Arthrobacter psychrolactophilus* B7 for industrial-scale D-tagatose with a half-life exceeding 1000 mins at 60 °C (Nirwantono et al. 2024), xylose (glucose) isomerases from hyperthermophilic *Thermotoga maritima* in the production of high-fructose corn syrup (HFCS) (Vieille et al. 2001), and recombinant xylosidase from *Clostridium clariflavum* in the biofuel synthesis (Zafar et al. 2022) are few examples that have demonstrated the need for thermostability in industrial focus applications.

The temperature at which the feather degradation process is carried out plays a critical role. Especially, the denaturation of recalcitrant proteins like keratin is a thermodynamically driven process. Elevated temperatures, firstly, augment the kinetic energy of molecular entities, thereby promoting the disruption of non-covalent interactions that are essential for preserving the protein’s native structural configuration. For instance, the thermostable pepsin at increased thermal conditions has shown to decrease the energetic threshold required for denaturation (Edelhoch 1960). Secondly, they facilitate the disruption of disulfide bonds within keratin, constituting an essential phase in its breakdown (Meng et al. 2022; Kanoksilapatham and Intagun 2017). Thirdly, they provide the requisite energy necessary to overcome the stabilizing interactions, like hydrogen bonding, hydrophobic interactions, and disulfide linkages, that contribute to the keratin’s complex structure, resulting in the disintegration of the protein’s conformation. Fourthly, proteins, including keratin, are susceptible to degradation mechanisms such as deamidation of asparagine and glutamine and succinamide formation at aspartate and glutamate residues (R. M. Daniel et al. 1996). To further substantiate, mesophilic *Streptomyces aureofaciens* K13 and *Bacillus cereus* VITSDVM4 produce keratinase with an activity of up to 44.2 U/ml and 174 U/ml, respectively (Cai et al. 2013; Subathra Devi et al. 2018), whereas thermostable keratinases from *Bacillus* sp. NFH5 and *Actinomadura keratinilytica* Cpt29 demonstrated activities of 1879.09 U/ml and 24,000 U/ml, respectively, which are significantly higher than typical mesophilic keratinase activities (Kokwe et al. 2023; Habbeche et al. 2014). Thus, high-temperature denaturation using thermostable keratinases might be critical in industrial processes for efficient degradation of feather proteins (Su et al. 2022).

The effective utilization of thermostable keratinases to operate under extreme industrial environments, characterized by elevated temperatures and fluctuating pH levels, renders them exceptionally appropriate. This inherent flexibility decreases the necessity for rigorous temperature regulation, thereby decreasing operational expenditures and energy utilization, and simplifying industrial processes (Parinayawanich et al. 2021). For instance, DgeKer, a thermostable keratinase from *Deinococcus geothermalis*, exhibits maximal activity at elevated temperatures (70 °C), which not only enhances their catalytic efficiency in breaking down keratin structures but also augmented the thermostability due to its extended half-life, which is a necessary prerequisite in the continuous feather degradation fermentation process (Tang et al. 2021).

For the sake of establishing an improved feather degradation system utilizing thermostable keratinases, it is necessary to study the biochemical and structural aspects of thermostable keratinases.

## Biochemical properties of thermostable keratinases

Bacterial thermostable keratinases afford a diverse array of biochemical characteristics, like stability under extreme conditions, substrate specificity, and catalytic efficiency, which are influenced by various factors such as temperature, pH, and the presence of metal ions or inhibitors, rendering them well-suited for a multitude of industrial and biotechnological purposes. The thermal stability of these keratinases that aid them to maintain their activity at elevated temperatures is a crucial aspect for industrial processes. For example, the keratinase produced by *Bacillus cereus* L10 demonstrates stability within the temperature parameters of 30 to 80 °C, maintaining an impressive 97.66% of its activity even at a temperature of 100 °C (Derhab et al. 2023). These enzymes also demonstrate peak enzymatic activity under alkaline environmental conditions that account for their pH stability. For instance, the keratinase derived from *Bacillus subtilis* NRC3 exhibited enhanced stability within a pH range of 5 to 10 (Tork et al. 2013). Certain thermostable keratinases possess the capability to hydrolyse a broad range of keratinaceous substrates. Accordingly, the keratinase from *Thermoactinomyces vulgaris* TK1-21 demonstrated the ability to degrade chicken feathers, pig bristles, and pig hooves, achieving significant hydrolysis rates and producing valuable keratin hydrolysates (Kaewsalud et al. 2023). The kinetic characteristics of these enzymes, including parameters such as *K*_*m*_ and *V*_max_, serve as indicators of their catalytic efficiency. For example, the keratinase derived from *Bacillus altitudinis* RBDV1 exhibits a *K*_*m*_ of 1.42 μM alongside a *V*_max_ of 1.673 μM/min, thereby implying substantial catalytic proficiency (Pawar et al. 2018). In addition, the presence of certain metal ions enhances the keratinase enzymatic activity. For instance, the enzymatic activity of *Bacillus subtilis* NRC3 keratinase activity is notably enhanced by the presence of metal ions such as Na^+^, K^+^, Mg^2+^, Ba^2+^, and Ca^2+^ (Tork et al. 2013). In contrast, certain thermostable keratinases exhibit inhibition in the presence of particular ions or chemical compounds. For example, the keratinase derived from *Bacillus* sp. CSK2 demonstrates inhibition when exposed to Al^3+^ and Fe^3+^, whereas its enzymatic activity is augmented by reducing agents such as 2-mercaptoethanol (Nnolim and Nwodo 2020).

## Structural characteristics of thermostable keratinases

1. MtaKer—*Meiothermus taiwanensis* WR-220

The thermophilic bacterium *Meiothermus taiwanensis* WR-220, obtained from Wu-rai hot springs in the northern part of Taiwan, has been identified as an effective feather-degrading bacteria that can complete feather decay at 65 °C, in 2 days via a 41.3 kDa extracellular monomeric serine protease (S8 family), MtaKer. The enzyme rMtaKer (recombinant form of MtaKer) maintains its keratinolytic activity across a wide temperature range from 25 to 75 °C, with optimal activity at 65 °C and also functions effectively in a broad pH range from 4 to 11, with peak activity at pH 10 (Wu et al. 2017).

**Fig. 3:**
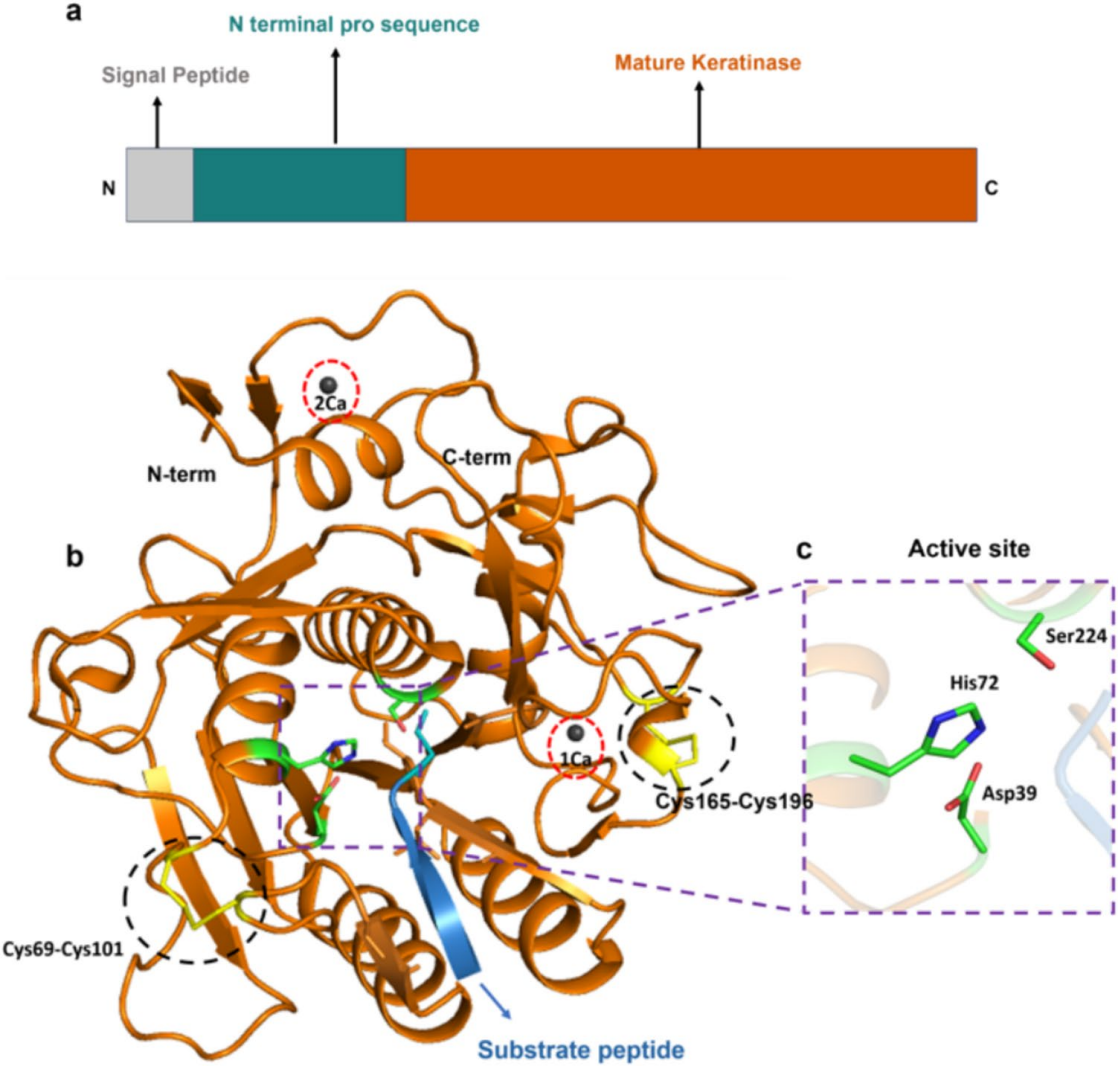
**a** Domain map of rMtaKer.** b** Crystal structure of mature rMtaKer (PDB ID:5WSL) represented in cartoon, purple dotted box showing the active site; red dotted circles show the two calcium ions (1 Ca and 2 Ca) depicted as black spheres, and black dotted circles indicate two intra-molecular disulfide bridges highlighted in yellow color, and the substrate C-terminal end of neighboring monomer (substrate) colored in marine blue **c** Close-up view of active site residues consisting of catalytic triad (Asp39, His72, Ser224)

The MtaKer keratinase sequence consists of a signal peptide at the N-terminus, an N-terminal propeptide (N-pro), and mature protease (Fig. 3a). The signal peptides facilitate the targeting of nascent proteins to the secretory pathway, whereas N-pro is a precursor to the enzyme that is excised to yield the enzyme functional for its proteolytic activity.

The monomer of mature rMtaKer (PDB ID: 5WSL) comprises of 16 β-sheets and 8 α-helices, with the central core composed of seven parallel β-sheets along with six α-helices (Fig. 3b), containing a conserved catalytic triad Asp39, His72, and Ser224, essential for peptide bond cleavage (Fig. 3c). In addition, the configuration of rMtaKer was also characterized not only by the presence of two calcium ions that are critical in ensuring proper folding and structural integrity but also by two intramolecular disulfide bridges that play a role in thermal stability and substrate recognition (red and black dotted circles in Fig. 3b). The primary calcium ion (1Ca) was stated to play a vital role in stabilizing the surface loop located between α1 and β2 by coordinating with Asp11 and Asp14, Asp11 and Thr23, Ser21, Gln15, and a water molecule, while adopting a pentagonal bipyramidal coordination geometry, while the second calcium ion (2Ca) interacts with the carbonyl oxygen atoms of the Val172, Gly175, and Thr177 residues in the main chain, as well as two water molecules. Whereas the initial disulfide bond, formed between Cys69 and Cys101, links the loops that play a part in substrate binding, and the second disulfide bond, formed between Cys165 and Cys196, was located in close proximity to both the 2Ca-binding site and the region containing residues of the active-site pocket (Wu et al. 2017) (Fig. 3b). These structural features enable the enzyme to remain stable at high temperatures and retain enhanced activity.

Furthermore, in the crystal structure (PDB 5WSL), C-terminal residues (^278^YEQLY^282^) of rMtaKer were found within the active-site cavity of a neighboring monomer, bringing the enzyme–substrate interaction (Fig. 3b). The interaction of the substrate (C-terminal residues) with the active site resulted in the transformation of the substrate loop into anti-parallel β sheet formation due to the interaction of substrate residues Asn280 and Leu281, with Ser104 and Gly105, of the substrate-binding cavity. Furthermore, the hydrophobic residues Ala156, Ala157, and Gly158 in the substrate-binding pocket of rMtaKer make hydrophobic interaction with the substrate, particularly with the residue Tyr282, of the substrate peptide chain (Wu et al. 2017). All these interactions stabilize the substrate-enzyme complex suitable for proteolysis.

2. Fervidolysin—*Fervidobacterium pennivorans*

**Fig. 4:**
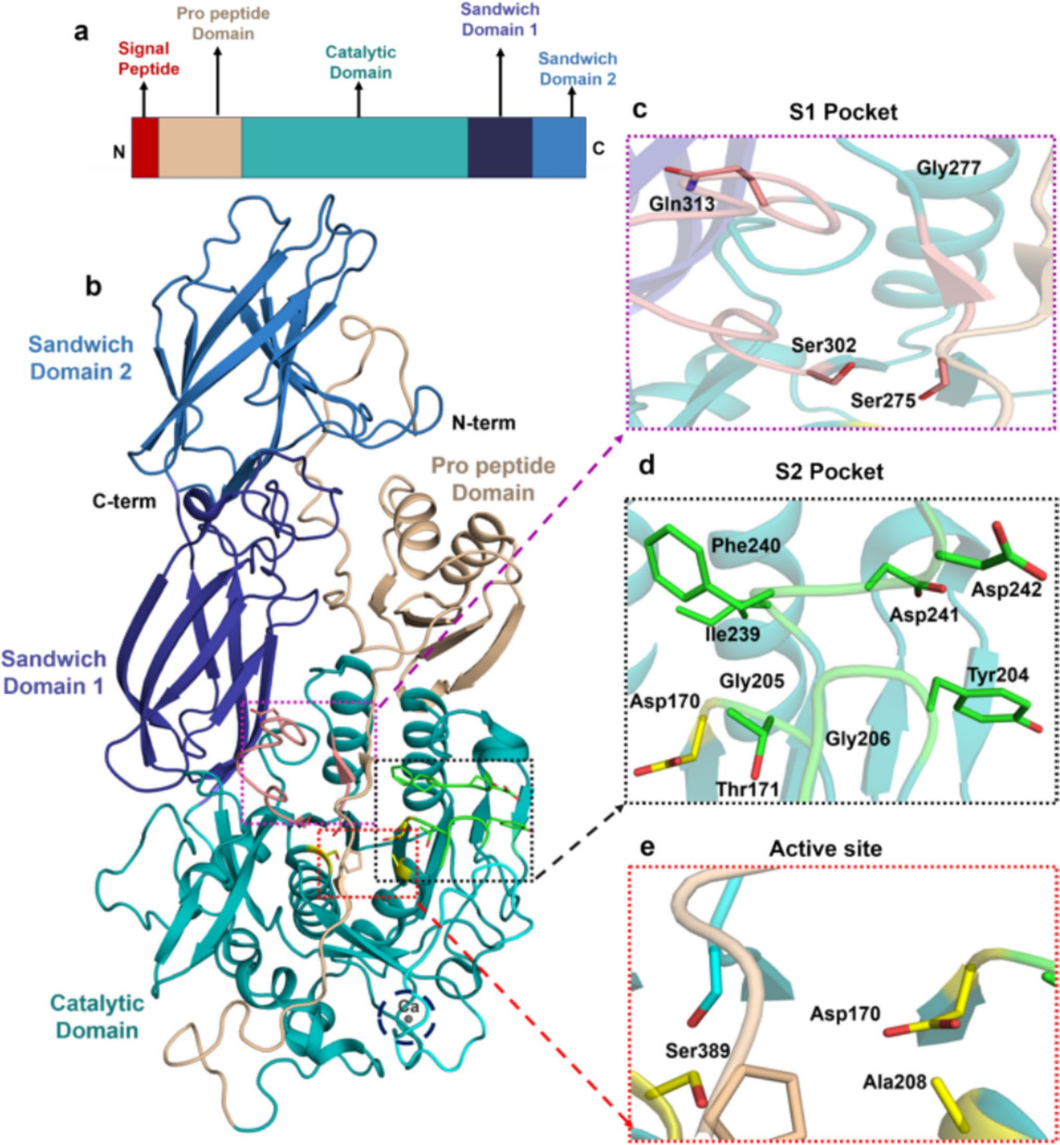
**a** Domain map of fervidolysin from *F. pennivorans* **b** Crystal structure of fervidolysin (PDB ID 1R6V) depicted in cartoon, with the pro-peptide domain (wheat colored), catalytic domain (cyan colored), and two sandwich domains (SD1-deep blue colored and SD2-marine colored calcium-binding site depicted by a grey sphere in a black dotted circle; S1 and S2 binding pockets shown in orange and black dotted boxes, respectively, along with the catalytic triad in a red dotted box. **c** Close-up view of S1 pocket. **d** Close-up view of S2 pocket.** e** Zoomed in view of the catalytic triad (Asp170, His208Ala, Ser389)

*Fervidobacterium pennivorans* is an extreme thermophile known to degrade feather at elevated temperatures, isolated from a hot spring in the Azores islands, thriving at extreme conditions such as 70 °C and pH 6.5 (Friedricht and Antranikian 1996). Fervidolysin is one among the several highly thermostable keratinases derived from *F. pennivorans*. Similar to MtaKer, fervidolysin also contains a signal peptide for extracellular localization, a pro-peptide domain (PD domain) for the enzyme’s maturation and activation, and a catalytic region (CD domain) primarily responsible for proteolysis (Kim et al. 2004) (Fig. 4a). In contrast to MtaKer, fervidolysin also contains additional two sandwich domains (SD1 and SD2) specifically involved in protein structural integrity (Kim et al. 2004) (Fig. 4a). The crystal structure of monomeric subtilisin-type serine protease, fervidolysin (PDB: 1R6V) (Fig. 4b), was in an inactive state due to mutation of one of the critical residues (His208Ala) (Fig. 4e) necessary for its activity.

Fervidolysin exhibited an elongated configuration with a distinct arrangement consisting of all four domains mentioned above (Fig. 4b). The N-terminal pro-peptide PD domain (Ser9-Asn128) had a globular conformation featuring elongated N- and C-terminal loop regions that extend across the molecule. The domain is stabilized by two α-helices and four β-strands that are interlinked through hydrophobic (Ile27, Phe29, and Val44) and hydrogen bond interactions. The N-terminal region of PD interacts with the SD2 domain, while the C-terminal part spans across the active site, connecting to the catalytic domain (CD) (Kim et al. 2004) (Fig. 4b).

Next, the catalytic domain (Ser129-Pro448) exhibits structural similarities to a subtilisin-like serine protease in protein folding. The salient features in CD include: first, the CD domain encompasses a catalytic triad consisting of three crucial amino acids: Ser389, His208 (mutated to Ala in this structure), and Asp170 (Fig. 4e); second, the CD domain, in contrast to subtilisin-like proteases, further comprises different insertions such as Asp242-Gly250 and Glu362-Gly379, which trigger localized structural alterations at the binding crevice (Fig. 4b). Third, the CD domain also consists of a hexa-coordinated (by six oxygen atoms) calcium-binding site (black dotted circle in Fig. 4b), crucial for the enzyme’s structure and function. Three of these oxygen atoms originate from the side chains of acidic residues, namely Glu137, Asp179, and Asp221. The remaining three oxygen atoms come from the peptide backbone, specifically from Lys219, Lys223, and Ile225, which appear proximal to the substrate-binding cavity (Fig. 4b). Fourth, CD domain exhibits unique substrate-binding sites, namely the S1 pocket and the S2 pocket. The S1 pocket (Fig. 4c) is partially defined by residues such as Ser275-Gly277 and Ser302-Gln313 whereas the S2 pocket (Fig. 4d) is constructed by residues Asp170-Thr171, Tyr204-Gly206, and Ile239-Asp242 (Kim et al. 2004).

Finally, fervidolysin has two unique β-sandwich domains (SDs), SD1 (Gly454-Asn563) and SD2 (Thr565-Gln679) at the C-terminus, which are not typically found in other subtilisin family proteins (Fig. 4b). They are composed of layers of β-sandwich sheets, contributing to its unique structural features and potentially its substrate interactions. While SD1, on the one hand, establishes polar interactions with the catalytic domain (CD), SD2, on the other hand, interacts with the N-terminal loop of the PD domain (Kim et al. 2004). These inter-domain interactions between SD1 and SD2 with other domains help in the overall enzyme’s structure, stability, and function (Kim et al. 2004) (Fig. 4b).

In summary, being a large protein (73 kDa) with four domains, the unique structural features of fervidolysin aided with stable inter-domain interactions enable efficient feather degradation at high temperatures. The individual roles of each domain and its effect on keratinolysis can be further explored in future through protein engineering and development of hybrid chimeras.

3. *Fi*BAP (Beta-aspartyl peptidase)—*Fervidobacterium islandicum* AW-1

*Fervidobacterium islandicum* is an extremely thermophilic anaerobic bacteria isolated from a hot spring near volcanic regions in Indonesia (Huber et al. 1990). The bacterium *F. islandicum* AW1 exhibits a diverse array of proteolytic enzymes, encompassing metalloproteases and peptidases, which demonstrate increased expression in response to nutrient deprivation and significantly contribute to keratin degradation (Kang et al. 2020). One notable enzyme within this category is the thermostable β-aspartyl peptidase (*Fi*BAP) derived from *Fervidobacterium islandicum* AW-1, which exhibits elevated expression during starvation that helps in the bacterium’s survival under stress conditions (Kang et al. 2020). Furthermore, *Fi*BAP plays a crucial role in the degradation of keratin via the hydrolytic cleavage of the peptide bond next to isoAspartic acid residues located at the C-terminus of the substrate peptide (La et al. 2020).

*Fi*BAP is identified as a 318 kDa octameric metallopeptidase, with its highest enzymatic activity observed at 80 °C and pH 7.0. *Fi*BAP activity was significantly increased in the presence of Zn^2+^, and Co^2+^was inhibited by 1% SDS and 1 mM EDTA (La et al. 2020).

The crystal structure of the enzyme (PDB: 7CDH) exists as an octamer consisting of a tetramer of dimers. The thermostability of the enzyme is greatly influenced by the interactions between the molecular subunits in the oligomeric state. Accordingly, the presence of an additional three salt bridges within each dimer resulted in a total of 12 ionic interactions, significantly boosting the enzyme’s impressive ability to withstand high temperatures (La et al. 2020).

**Fig. 5:**
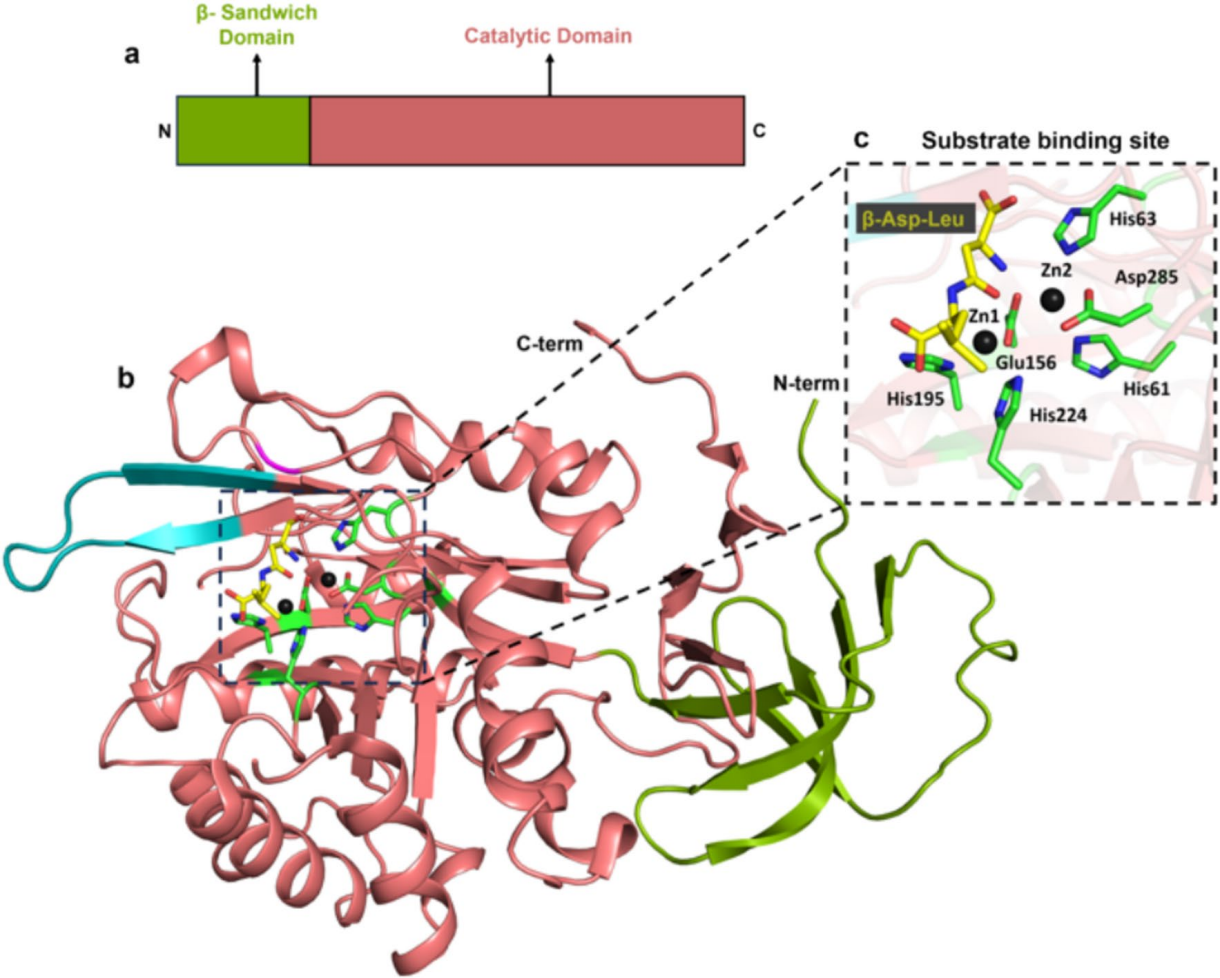
**a** Domain map of *Fi*BAP. **b** Crystal structure of *Fi*Bap with ligand-bound form (PDB ID: 7CF6) represented in cartoon model with the β-sandwich domain and catalytic domain shown in smudge green and salmon, respectively. The black dotted box depicts the substrate binding site with two zinc ions as black spheres, coordinated by catalytic residues shown in green, and the substrate β-Asp-Leu in the yellow stick model. The transformed loop (Pro290-Leu302) into two antiparallel β-sheets upon substrate binding, shown in cyan. **c** Close-up view of substrate-binding site with two zinc ions in black spheres, coordinated by surrounding active site residues (green) denoted by sticks and substrate β-Asp-Leu in yellow

The monomeric protein *Fi*BAP structure comprises an N-terminal β-sandwich domain (Met1–Gly56; Gly344–Glu386) and a C-terminal catalytic domain (Leu57–Lys343) responsible for dimerization and enzyme activity, respectively (Fig. 5a). The β-sandwich domain consists of nine β-sheets organized into two layers, with β1, β3, β4, and β5 forming one layer and β2, β6, and β17–β19 forming the second layer, that provide stability to the overall structure (Fig. 5b). The catalytic domain adopts a (β/α)_8_ triosephosphate isomerase (TIM)-barrel structure, consisting of eight β-sheets and nine α-helices (α2–α10) (Fig. 5b). This structure encloses the central core, which houses the substrate-binding site referred to as the binuclear zinc centre, constituted by two Zn^2+^ ions (black dotted box in Fig. 5b). Zn1 is coordinated by the residues Glu156, H195, and His224, whereas Zn2 is bound by His61, His63, Glu156, and Asp285. Within the metal coordination network, Zn1 interacts with Zn2 through Glu156 (La et al. 2020) (Fig. 5c). Another interesting structural aspect of this enzyme is that, in the ligand-bound form of the protein bound with a substrate analogue, N-carbobenzoxy-β-Asp-Leu (PDB ID: 7CF6) (yellow colored stick in Fig. 5c), loop Pro290-Leu302, which was disordered in apo-form, transformed into two stable antiparallel β-sheets (β14 and β15) as a result of ligand binding (colored cyan in Fig. 5b). The proximity of this loop to the substrate-binding site suggests its role in regulating substrate entry and product exit (La et al. 2020). The substrate analogue is coordinated by residues such as Tyr130, Gly68, and Ser289, which are situated in closer proximity to the ligand compared to their arrangement in the ligand-free conformation, thereby facilitating both coordination and stabilization (Fig. 5c).

The above mentioned structural features of *Fi*BAP, such as, its structural fold enable efficient binuclear Zn atom-mediated keratinolysis at high temperature. Furthermore, its octameric state further provides stability to the overall protein. The enzyme’s specificity towards iso-aspartyl residues for proteolysis can be exploited further in the development of feather degradation systems.

4. *Fis*CP (M32 Carboxypeptidase—*Fervidobacterium islandicum* AW-1

*Fi*sCP is a metal-dependent protease enzyme encoded by the FIAW1_1600 gene in *Fervidobacterium islandicum* AW-1, which belongs to the M32 carboxypeptidase family and cleaves C-terminal amino acid residue from proteins and peptides. *Fi*sCP is identified as a 107 kDa dimeric protease capable of degrading feathers at high temperatures of 80 °C and a neutral pH of 7.0 (Lee et al. 2015a, b).

**Fig. 6:**
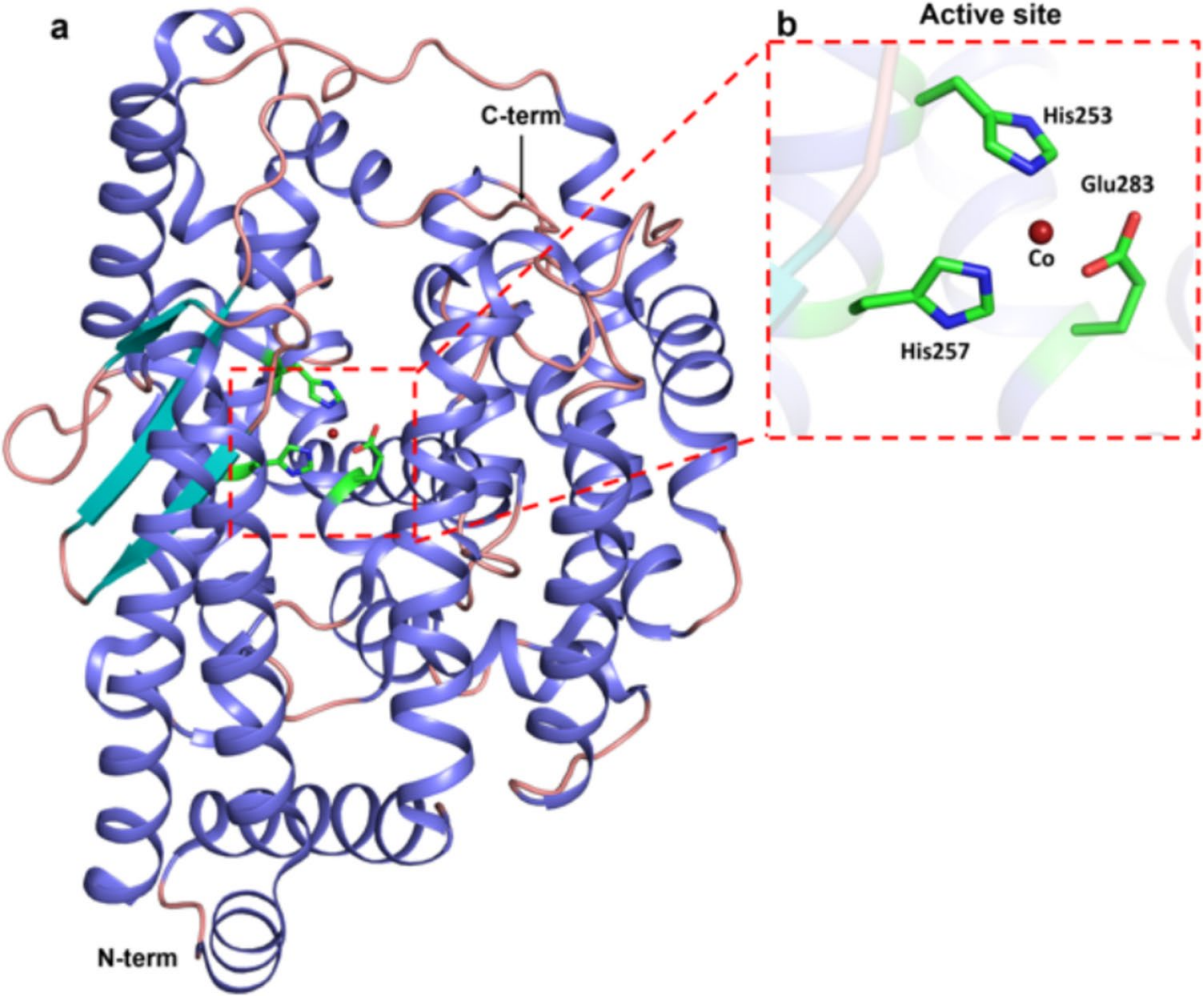
**a** Crystal structure of *Fi*scp (PDB ID 5E3X) represented in cartoon with helices, β-sheets, and loops represented in slate, cyan, and salmon, respectively. The red dotted box depicts the active site with the Co^2+^ ion represented as a firebrick red sphere. **b** Close-up view of active site with Co^2+^ as firebrick red sphere with surrounding residues shown as sticks (His253, His257, and His283)

The crystal structure of *Fi*sCP (PDB: 5E3X) demonstrates a predominantly helical form, with a three-stranded β-sheet (colored cyan in Fig. 5a) located next to the active site (Fig. 6a). The oligomerization and the structural fold of the enzyme appear to be crucial for the enzyme’s function, allowing it to interact with substrates and cause hydrolysis of the peptide bond. The oligomeric state is bolstered by the presence of hydrophobic interactions, salt bridges, and hydrogen bonds between interfacial residues of the facing monomers, which are crucial for its thermostability (Lee et al. 2015a, b). The active site (red dotted box in Fig. 6a) is located within a substrate-binding groove of *Fi*sCP that contains the residues from the characteristic HEXXH motif of M32 carboxypeptidase and a Co^2+^ binding site. The metal is coordinated by residues His253, His257, and Glu283 (Fig. 6b). This coordination is essential for the enzyme’s stability and activity. The opening and closing of the substrate groove due to hinge-bending motion are necessary for the entry and exit of the substrate into the active site (Okai et al. 2017). Unlike TthCP (PDB: 3HOA), which has a substrate groove in a closed state, the structure of *Fi*sCP exists in an open state. Peptide substrates are proposed to enter through this groove, where their C-terminal carboxylate group coordinates with Arg335 and Tyr407 of *Fi*sCP (Fig. 6a). Upon reaching the active site (Fig. 6b), the acid–base reaction involving the critical residues and the metal ion facilitates the peptide bond cleavage (Lee et al. 2015a, b).

To sum up, the structural features containing the prevalent alpha-helical region, aided by the metal-mediated carboxypeptidase activity of the *Fi*sCP at the active site, and the flexible substrate-binding groove give rise to enhanced hydrolysis of keratin peptide fragment.

5. *Fi*SufS-SufU—*Fervidobacterium islandicum* AW-1

*Fervidobacterium islandicum* AW-1 possesses a fully fledged Suf-like machinery (SufCBDSU) that is significantly upregulated when cultured on native feathers lacking elemental sulfur (S^0^) (Kang et al. 2020). Especially, the SufS and SufU proteins from *Fervidobacterium islandicum* AW-1 exhibit cooperative sulfur transfer activity through the recycling of cysteine and cystine, which are essential for maintaining redox homeostasis within cellular environments by facilitating the export of excess cysteine. Consequently, provoking the oxidative stress, thus aiding in the formation of extracellular reducing conditions and promoting the breakdown of feather keratin (Jin et al. 2021). Furthermore, the thermodynamically driven feather degradation rate is driven forward by efficient utilization of the released cysteine residues, the end product of keratinolysis. Thus, the Suf system is specifically upregulated in response to keratin-rich environments, and the addition of the *Fi*SufS-SufU complex to cell extracts increased the rate of feather decomposition (Jin et al. 2021).

In general, the Suf machinery in bacteria encompasses the SufS-SufU complex, which is integral to the biosynthesis of iron–sulfur (Fe–S) clusters and is pivotal for numerous cellular processes like respiration, nitrogen fixation, electron transfer, and redox catalysis. The SufS enzyme catalyzes the de-sulfurization of L-cysteine, subsequently transferring the sulfur to the SufBCD scaffold through SufU, which, in turn, promotes the assembly of iron–sulfur (Fe–S) clusters (Singh et al. 2013). Therefore, the Suf system exhibits elevated expression levels in response to oxidative stress or iron deficiency in bacteria, which can aid in effectively sustaining Fe–S homeostasis and fostering cellular growth under adverse conditions (Blahut et al. 2020).

**Fig. 7:**
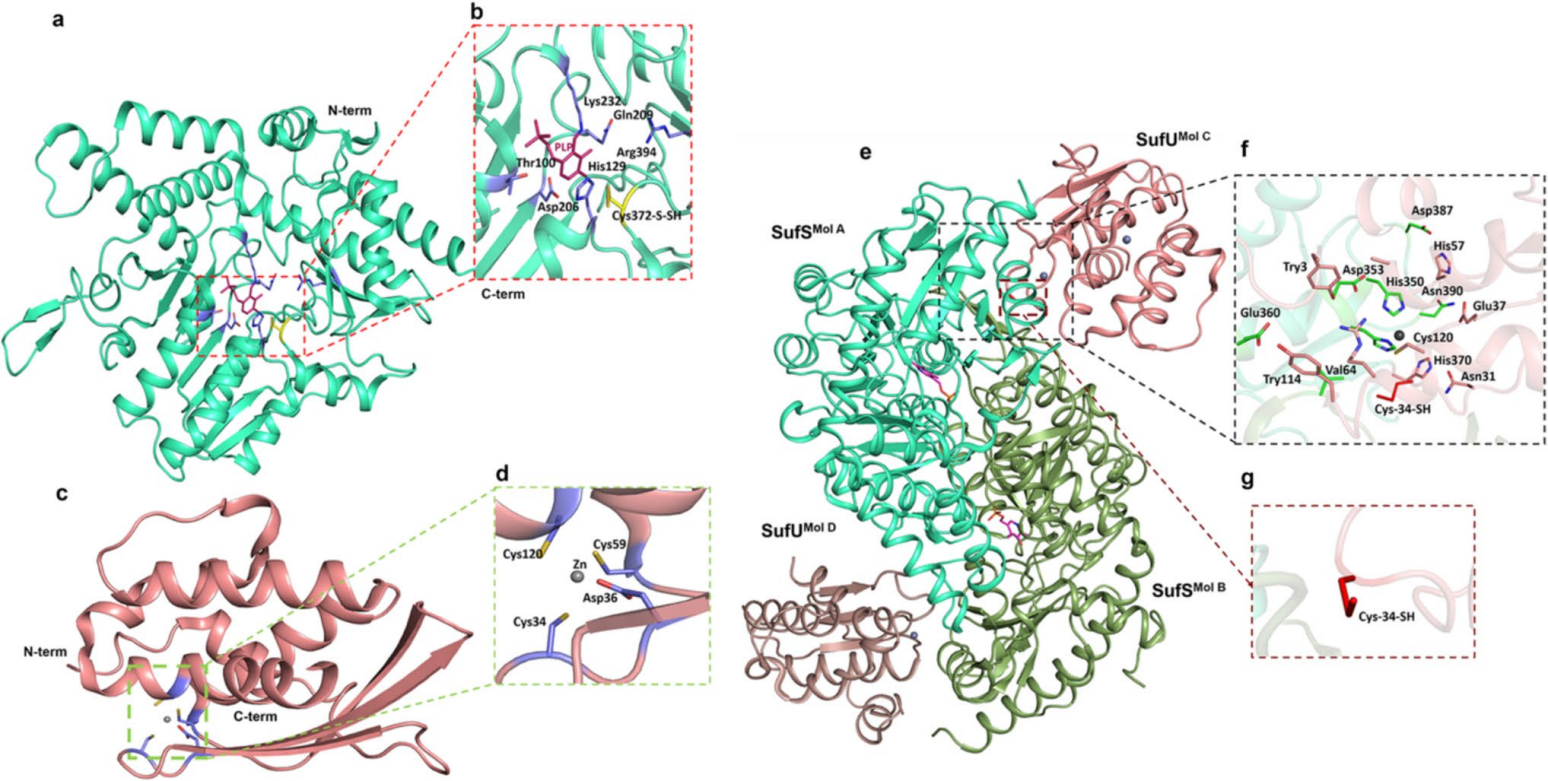
**a** Crystal structure of *Fi*SufS (PDB ID 6A6E) represented in cartoon with the active site containing PLP and surrounding residues depicted in red dotted box **b** Close-up view of active site with active site residues in sticks, co-factor PLP in magenta color, and Cys372-S-SH involved in sulfur transfer in yellow color **c** Crystal structure of *Fi*SufU (PDB ID: 6A6F) depicted in cartoon with green dotted box showing the active site **d** Zoom-out view of the active site containing Zn and critical residues. **e** Crystal structure of the *Fi*SufS-SufU complex (PDB ID 6A6G) is represented in cartoon with each monomeric unit colored distinctly and labelled. The black dotted box represents the active site and critical residues; the brown dotted box shows Cys34-SH, critical for sulfur transfer. **f** Close-up view of the residues between *Fi*SufS and *Fi*SufU at the interface. **g** Zoomed in view of Cys34-S-SH that undergoes structural modifications upon complex formation

The *Fi*SufS (PDB ID: 6A6E) is a homo-dimeric protein with an apparent molecular mass of 94 kDa. The crystal structure of the protein contains 14 α-helices and 12 β-sheets (Fig. 7a). The active site of the enzyme consists of Lys232 covalently bound to the cofactor pyridoxal phosphate (PLP), for desulfurization reaction, and per-sulfurated Cys372-SH, which is crucial for the sulfur transfer process (Fig. 7b), whereas *Fi*SufU (PDB ID: 6A6F) is a 15.5 kDa monomeric protein that consists of 6 α-helices and 3 β-sheets (Fig. 7c) with a Zn ion in the active site coordinated by Asp36, Cys120, Cys34, and Cys59 (Fig. 7d). The crystal structure of the *Fi*SufS-SufU complex (PDB ID: 6A6G) (Fig. 7e) revealed specific protein–protein interactions that facilitate the sulfur transfer process (Jin et al. 2021). Upon *Fi*SufU’s complex formation with *Fi*SufS, structural changes in *Fi*SufU enhance its ability to receive sulfur from *Fi*SufS. First, two β-sheets containing Cys34-S-SH in *Fi*SufU were significantly reduced in length and were transformed into a loop, while the length of the loop containing Cys34 increased, offering flexibility for loop movement (Fig. 7e and f). Next, the loop containing Cys34 translocated into the groove of *Fi*SufS, which is causing the sulfur transfer from SufS to SufU (Fig. 7g) (Jin et al. 2021).

With the addition of the *Fi*SufS-SufU enzyme in the feather degradation system, the keratinolysis reaction can be fastened as it drives the forward reaction with the continuous utilization of the cysteine end product. This is evident with the finding that the addition of the *Fi*SufS-SufU complex along with whole cell lysates increased the rate of feather decomposition in vitro (Jin et al. 2021). Although the *Fi*SufS-SufU system favors sulfitolysis of feathers in FDB through the cysteine-cystine cycle, it can enhance the rate of sulfitolysis only when it is mixed with whole-cell FDB extract or other keratinases (Jin et al. 2021). Therefore, this thermostable enzyme complex can also be added in the feather degradation system along with other keratinases to improve the enzymatic feather degradation process.

## Protein engineering: improvement of microbial keratinases

Protein engineering is one of the biotechnological tools for creating newer protein structures by altering the genes to improve their biological activity. This method to create proteins with enhanced natural behavior needs the fundamentals of the structure–function relationship as a prerequisite. Consistently, the application of protein engineering in the field of enzymatic keratinolysis has been performed to enhance not only the keratinolytic activity but also its thermostability. For instance, the flexible loop in keratinase KerZ1 from thermophilic *Bacillus licheniformis* was engineered to be more hydrophobic and rigid, resulting in its enhanced thermal stability (Peng et al. 2022). In the same vein, the S1 substrate-binding pocket of a thermally stable keratinase 4-3Ker from *Pseudomonas aeruginosa* 4–3 was engineered to improve its substrate specificity and catalytic activity (Pei et al. 2024). Such modifications in structural elements result in enhanced degradation velocities and elevated enzyme efficacy under industrially relevant conditions.

Furthermore, several other genetic engineering approaches, such as mutations, directed evolution, homologous recombination, and gene expression manipulation, have been employed to engineer keratinases. Firstly, the incorporation of non-canonical amino acids (ncAAs) in place of regular amino acids such as *p*-benzoyl phenylalanine (*p*BpF) for Phe, in the active sites improved the catalytic activity of enzymes (Pagar et al. 2022). Accordingly, the keratinase derived from *Pseudomonas aeruginosa* (KerPA) was engineered to have the non-canonical amino acid *p*BpF in place of Tyr21, Tyr70, and Tyr114, which led to a protein engineered variant with improved efficacy and stability, notably exhibiting a 1.3-fold enhancement in keratinolytic activity and an 8.2-fold extension in half-life under reducing circumstances (Pan et al. 2021). Secondly, directed evolution technology involves iterative rounds of mutagenesis and selection to evolve proteins with desired traits. For instance, directed evolution of keratinase enzyme kerBp from *Brevibacillus parabrevis* specifically via error-prone PCR generated nine mutants that exhibited enhanced keratinase activity. Out of nine mutants, the most effective mutant increased enzyme activity from 1150 to 8448 U/mL and improved the feather degradation rate from 49 to 88% (Zhang et al. 2022).

Thirdly, by applying site-directed mutagenesis, keratinase (4-3Ker) from *Pseudomonas aeruginosa* 4–3 utilized rational engineering of the S1 substrate-binding pocket to enhance catalytic activity and substrate specificity via mutations M128R, A138V, and V142I. Further, the M128R/A138V/V142I triple mutant exhibited a 1.21-fold increase in keratin catalytic activity and enhanced thermal stability, resulting in a 32.86% improvement in feather degradation relative to the wild-type (Pei et al. 2024). Similarly, the thermostability and catalytic efficiency of the keratinase from *Bacillus licheniformis* BBE11-1 were improved through the incorporation of four amino acid substitutions (N122Y, N217S, A193P, and N160C), which resulted in a significant 8.6-fold extension of the half-life at 60 °C and a substantial 5.6-fold enhancement in catalytic efficiency (Liu et al. 2013). Finally, strategy to build hybrid keratinases by domain exchange between two closely related enzymes was also implemented. For instance, the substitution of the C-terminal domain of keratinase KerSMD from *Stenotrophomonas* sp. with that of its homolog KerSMF improved keratinolytic activity and increased the catalytic efficiency (*k*_cat_/*K*_*m*_) by 54.5% on a synthetic peptide. However, the combined replacement of both N- and C-terminal domains led to a more stable enzyme variant, with a melting temperature (*T*_*m*_) of 64.60 ± 0.65 °C and a half-life of 244.6 ± 2 min at 60 °C, indicating enhanced thermostability. This hybrid variant displayed over a twofold rise in catalytic efficacy and enhanced heat resistance than the native keratinases (Fang et al. 2016).

All these instances demonstrate undoubtedly the critical role of protein engineering in enhancing the current keratinolytic enzymes. Thus, similar strategies, if employed, to the identified thermostable keratinases might decrease the time taken for large-scale feather degradation.

## Applications of thermostable microbial keratinases

**Fig. 8:**
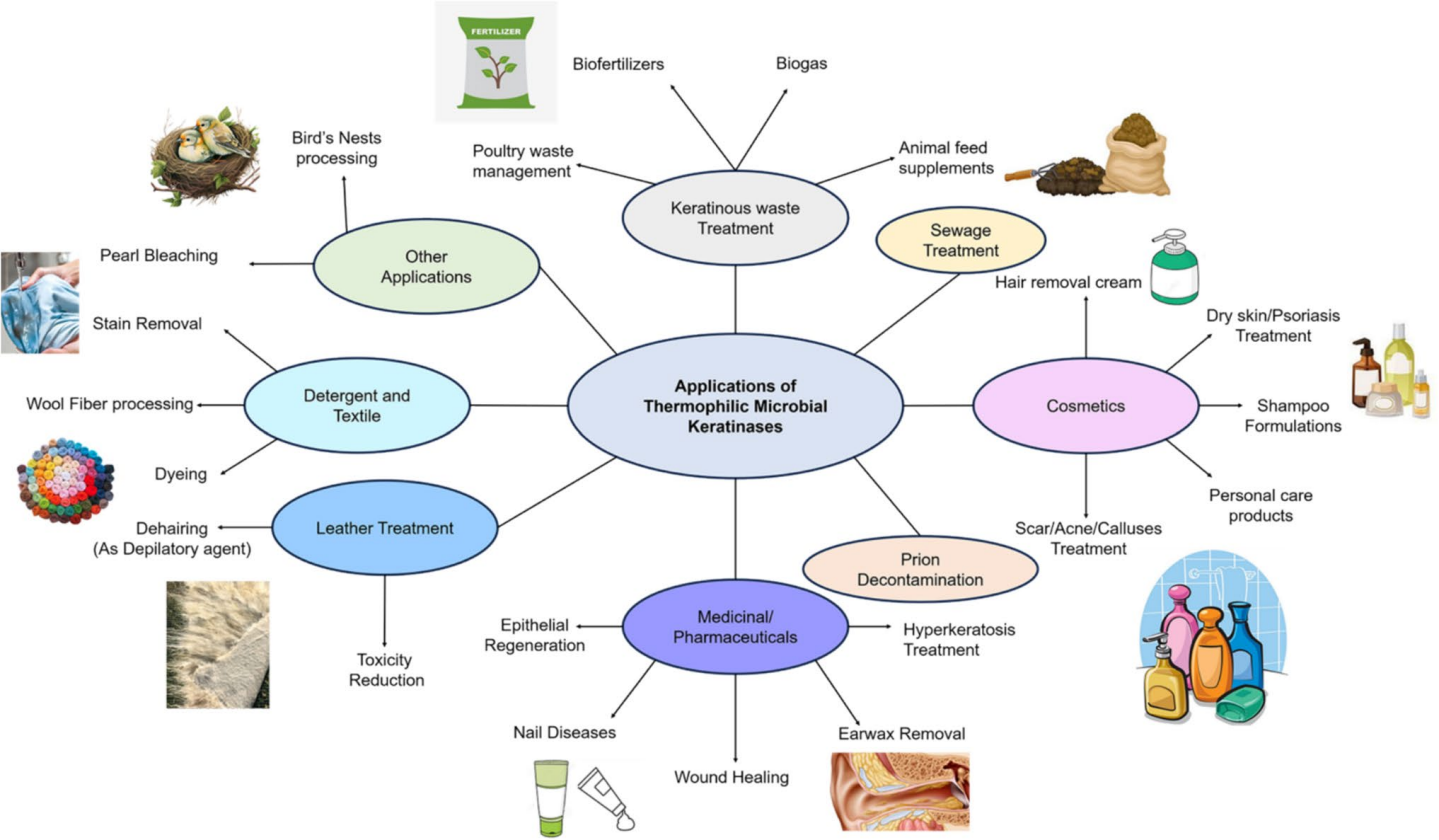
Applications of thermostable bacterial keratinases

Thermostable microbial keratinolytic enzymes demonstrate considerable potential in the eco-friendly degradation of keratin-rich feather waste, providing an environmentally sustainable and efficient approach to managing their accumulation. The potential applications of thermostable keratinases are vast, encompassing a range of industries including agriculture, animal nutrition, cosmetics, pharmaceuticals, and waste management (Vidmar et al. 2018; de Menezes et al. 2021) (Fig. 8). In the leather and textile sectors, keratinases are employed as environmentally sustainable dehairing agents, yielding high-quality products characterized by smooth and flexible surfaces (Derhab et al. 2023; Akram et al. 2020; Esmail et al. 2024). Whereas, in the detergent sector, keratinases are integrated into laundry detergents due to their efficacy in degrading proteinaceous stains such as blood and egg albumin (Derhab et al. 2023). For instance, thermostable keratinases derived from *Bacillus* sp. NKSP-7, *Bacillus aerius* NSMk2, and *Bacillus altitudinis* RBDV1 have demonstrated remarkable dehairing efficiency and are also utilized in laundry detergent formulations (Akram et al. 2020; Bhari et al. 2019; Pawar et al. 2018). As a fertilizer, the feather hydrolysate produced by thermostable bacteria such as *Bacillus cereus* L10’s keratinase has been shown to enhance plant growth, acting as biofertilizers that improve seed germination and overall plant development (Derhab et al. 2023; de Menezes et al. 2021).

Towards energy sustainability, the transformation of keratinous biomass into biofuels represents an additional promising application of thermostable keratinases. The feather hydrolysates generated by keratinases can be subsequently used as a substrate for biofuel production during fermentation, contributing to energy sustainability and mitigating dependence on fossil fuel sources (Mazhar et al. 2023). Accordingly, chicken feathers pretreated by *Bacillus* sp. C4 were successfully used as a substrate for methane gas production (de Menezes et al. 2021) (Fig. 8).

Within the medical domain, keratinases are utilized for the degradation of prions and the removal of human calluses and are incorporated into various dermatological pharmaceuticals and cosmetic formulations (Esmail et al. 2024). Moreover, the enzyme’s potential applications in drug delivery systems are currently under investigation, wherein keratin-derived biopolymers may function as carriers for therapeutic agents (Mazhar et al. 2023).

Thus, the thermostable keratinase has a wide scope of applications, from the hydrolysates being used as an animal feed to medicine in dermatological and cosmetic formulations (Fig. 8), inviting research towards re-engineering keratinases in the next few decades.

## Summary and conclusion

Feather wastes from poultry industries tend to remain a major environmental pollutant due to their resistance to natural biodegradation. Feather degradation is critically affected due to the numerous disulfide bonds present in the keratin protein, its major constituent. Therefore, incineration, landfill dump, and chemical acid treatment were used as a primary waste management process (Fig. 1). In contrast to these nutrient-depleting methods of feather waste treatment, microbial keratinolysis was identified as an eco-friendly alternative to retain its nutrient content and recycle the hydrolysates as nutrient feed (Table 1). The persisting problem, such as slower rate of microbial feather degradation, was overcome by enzymatic keratinolysis, where the enzymes that degrade feather were identified (Table 2) and are recombinantly overexpressed to degrade feather in vitro under optimal conditions in an industrial setting. The higher temperatures attained in waste treatment plants in industrial settings became a limiting factor for the keratinases derived from mesophiles. To overcome this, thermostable bacterial keratinases from thermophiles appear to be more efficient in fastening the thermodynamically driven feather degradation process. The feather degradation involves first the reduction of disulfide bridges (sulfitolysis) in feather, followed by proteolysis to peptides by exo-peptidases and keratinases, and finally the amino acid generation from the cleaved peptides using endo-peptidases (Fig. 2).

This review has examined the complex structural attributes of five thermostable microbial keratinases: firstly, MtaKer from *Meiothermus taiwanensis* WR-220 (Fig. 3) and fervidolysin from *F. pennivorans* (Fig. 4) capable of converting keratin substrates into peptide fragments via peptide bond hydrolysis. Secondly, beta-aspartyl peptidase (*Fi*BAP) from *F. islandicum* (Fig. 5) and *Fi*sCP (M32 carboxypeptidase) from *F. islandicum* AW1 (Fig. 6), involved in the conversion of peptide fragments into smaller amino acid end products. Finally, the SufS-SufU complex system from *F. islandicum* (Fig. 7) that is indirectly involved in the sulfitolysis of disulfide bonds in keratin under extracellular reducing conditions causes the conversion of cystine into cysteine and S-sulfocysteine, further making the proteins accessible to hydrolysis by various proteases.

In summary, the comprehension of feather degradation processes in nature opens a way to exploit them in a large industrial scale for efficient degradation of accumulated several tonnes of feather pollutants. One of the plausible means towards an efficient feather degradation system is to identify a rational combination of these identified thermostable keratinases that specialize in sulfitolysis, peptide hydrolysis, and amino acid generation, that could significantly improve the overall feather degradation rate through a step-wise degradation process ex vivo. In addition, the structures of keratinases elaborated in this review can be protein engineered to enhance the efficiency of degradation, which further requires experimental validations. Such efficient thermophilic microbial keratinases cannot only be restricted to feather pollutant degradation, but also be utilized for a wide range of industrial applications, from waste treatment to medical and cosmetic formulation preparations (Fig. 8).

The current review integrates the structural comprehension of known thermostable keratinases with their practical applications with respect to the growing industrial demand for thermostable enzymes. Thereby, thermostable bacterial keratinases-mediated feather pollutant degradation presents a promising, eco-friendly solution within the central framework of pollutant degradation via microbial consortia.

## Data Availability

No datasets were generated or analysed during the current study.
